# Impacts on insulation resistance of thin film modules: A case study of a flooding of a photovoltaic power plant in Thailand

**DOI:** 10.1371/journal.pone.0274839

**Published:** 2022-09-19

**Authors:** Nipon Ketjoy, Pornthip Mensin, Wisut Chamsa-ard

**Affiliations:** School of Renewable Energy and Smart Grid Technology, Naresuan University, Phitsanulok, Thailand; China University of Mining and Technology, CHINA

## Abstract

Effects of high humid weather conditions on photovoltaic (PV) modules were examined in this study, particularly insulation resistance. Three types of tests were conducted which include leakage voltage test, leakage current test, and wet leakage current test. Due to the usual field constraints in the study of insulation resistance, which limited measurements of leakage current, assurance was made that representative sampling were conducted. The study found a high number of modules with low insulation resistance and high leakage voltage values, which can interrupt the PV plant operation. High leakage voltage creates safety hazards issues. About two third of the samples, which showed deep moisture ingress in the modules, could not pass the minimum criterion of IEC 61646 standard for wet insulation resistance testing. The leakage current results showed the same trend as of leakage voltage, proving that leakage voltage test, which is quite easy and economical, can be used to detect such type of problems in field tests. Prolonged humidity conditions of the PV power plant particularly from natural disaster, should be avoided. Efficient drainage system should be supported in and around installations and all other moisture sources should be regularly removed from the plant area to keep proper operation and minimize losses in energy production.

## Introduction

**Fig 1:**
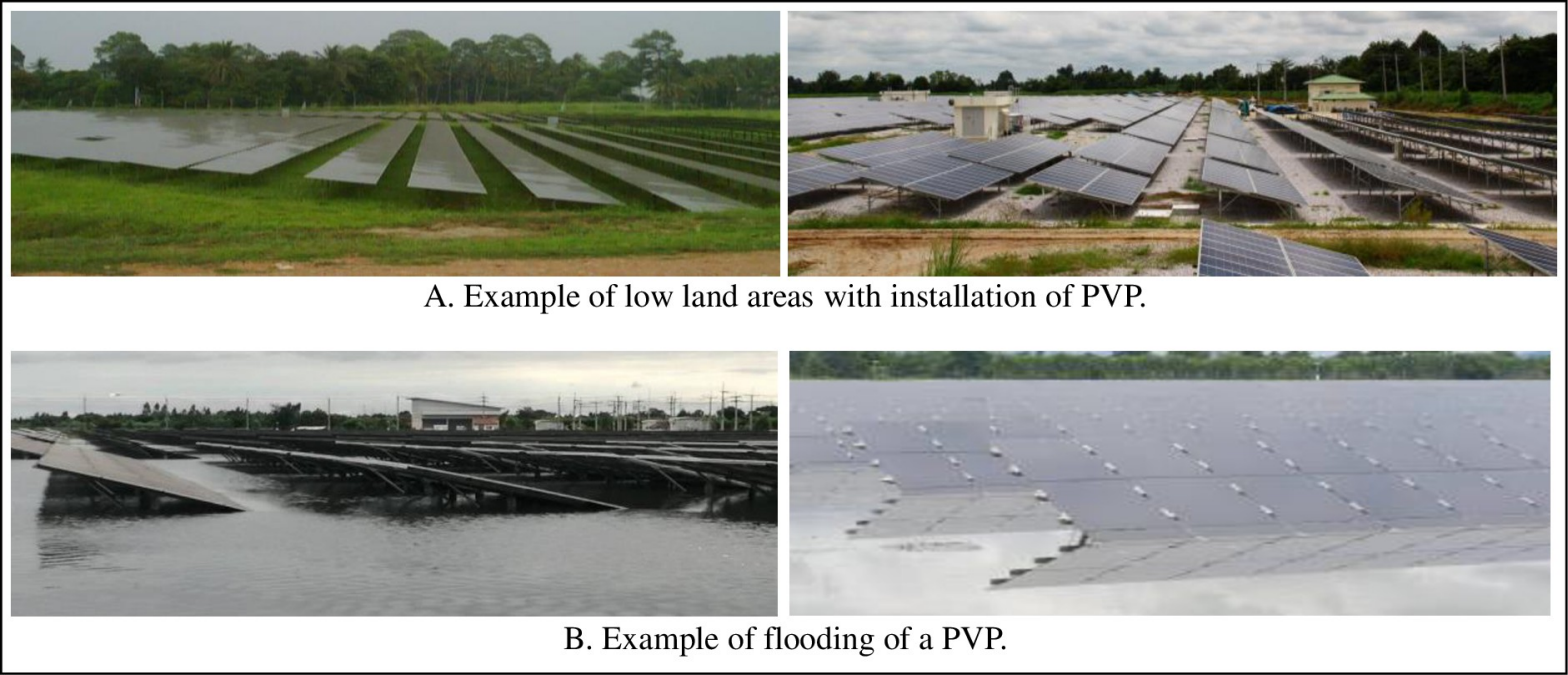
PVP in Thailand. A. Example of low land areas with installation of PVP. B. Example of flooding of a PVP.

During the last fifteen years many grid-connected photovoltaic power (PVP) plants have been established in Thailand in response to the “Adder” and FIT (Feed-in tariff)” programs, which are financial support programs offered by the government to increase deployment of renewable energy [1, 2]. These programs led to a preference for ground mounted PVP plants or what are now commonly called “solar PV farms”. Many “solar PV farms” are in low land areas that are affected by flooding (Fig 1). Flooding of these PVP plants leads to operational shutdown, production losses, and asset losses. Thailand has high humid weather conditions. The rainy season on average lasts for almost five months and there is frequent flooding in many parts of the country during the rainy season. There is a need to study the effects of the exposure of PVP systems to extremely high humid conditions, particularly submersion in floodwaters.

### Climate conditions in Thailand

**Fig 2:**
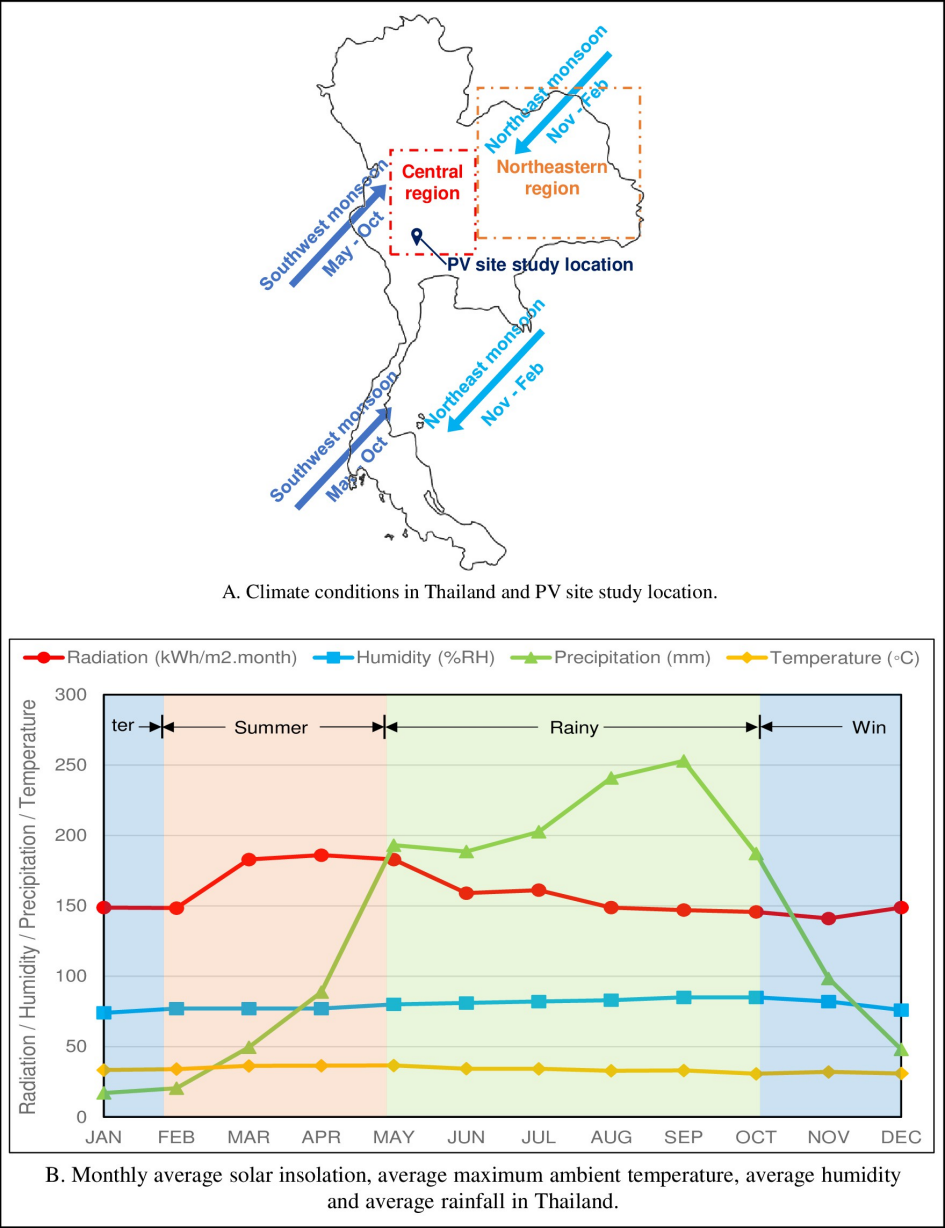
Overall climate conditions of the country and regions of highest solar radiation. A. Climate conditions in Thailand and PV site study location. B. Monthly average solar insolation, average maximum ambient temperature, average humidity, and average rainfall in Thailand. The precipitation is average monthly rainfall during 30-year period [3–6].

Thailand has an annual average solar insolation of 5.06 kWh/m^2^.day, with 50% of the total area of the country receiving an annual average solar radiation of between 5.00–5.28 kWh/m^2^.day. The areas that receive the highest amount of solar radiation are the Northeastern and Central regions (Fig 2A) [3]. Monthly average of solar radiation is quite constant throughout the year except in summer season when it is 15% higher compared to the annual average (see Fig 2B red line). With this high solar radiation potential, solar PV farm projects have been installed in all regions of the country, especially in the two regions.

Thailand is in the tropical zone [4]. From November until February, the country is affected by the northeast monsoon, inducing cool and dry air over major parts of country. The period of middle-October to middle-February is considered the winter season. During winter, rainfall in the rest of the country is much less, with December and January usually the driest months. Upper Thailand has cool and dry weather. During these months, eastern winds bring high rainfall only to the east coast of Thailand southern peninsula. From mid-May to mid-October, the country is subjected to the southwest monsoon, which bring high rainfall to the whole of the country. These months of the southwest monsoon is the rainy season. Within August to October, rainfall peak brought by tropical cyclones. Many parts of the country get flooded during this time.

Fig 2B (S1 Data) shows the monthly mean rainfall pattern in the country (i.e., the green line) which is influenced mainly by the southwest and northeast monsoons as described above. It is normally cloudy during the rainy season, one reason solar radiation during this period is relatively low compared to summer season. Summer season is from middle February to middle May, with April the hottest month [4, 7].

There is direct relationship between ambient humidity and rainfall, and the annual average RH (Relative Humidity) is 80%, which show that PV modules and other electronic devices (especially inverters) of solar PVP plants are always operated under high humid condition even during the dry season.

### Effects of humid conditions on PV modules

PV modules delamination failures happened often even in moderate climate environments as reported by Poulek et al. [8] in a study done in the Czech Republic. These were mostly edge delamination defects that possibly occurred during standard module fabrication. There is also edge delamination due to the penetration of moisture into the modules because of high humidity and rainfall, and this can cause major PV module and inverter failures as it can lead to high string voltage causing an electric discharge channel between the string of PV modules and the grounded module frames. Thus, water ingression or penetration (because of high humidity) can be a major cause of failures.

However, the degradation of the PV modules is highly affected not only by climatic conditions but also by the type of materials used for the modules. Omazic et al. [9] have studied: the degradation of the PV modules looking at the relationship between the polymeric components of the PV modules and climatic conditions, an overview and explanation of the pattern of external stresses (i.e., climate effects especially temperature, humidity and rain cycles that are affecting module performance and lifetime) and internal stresses (i.e., from failures induced by the polymeric materials) on the PV modules. The study showed that hot and humid tropical climates cause a more rapid and serious degradation of PV modules compared with other climatic conditions. The study clearly showed that the delamination of PV modules is more frequent and serious under tropical condition (which is consistent with the study of Poulek et al., mentioned above).

### Impacts on PVP systems in Thailand

Thailand being located near the equator and with a tropical environment has hot and highly humid weather conditions. The rainy season on the average lasts for almost five months (the average precipitation within a 30-year period is about 1,600 mm/year). There is frequent flooding in many parts of the country during the rainy season.

Many PVP plants, such as the solar PV farms, are in low land areas that are affected by flooding. Flooding of solar PV farms leads to operational shutdown, production losses, and asset losses. Flooding can submerge and damage PVP components such as PV modules, DC/AC combiner boxes, inverters, and transformers. Given the results of the two studies mentioned in the previous sub-section, it is especially important to evaluate the effects of flooding, particularly on the PV modules. There is a need for a study in Thailand to investigate the extent of module degradation and failures in insulation resistance after the exposure of PVP systems, specifically solar PV farms, to extremely high humid conditions, particularly submersion in floodwaters.

## Objective of the study

The aim of this study was to evaluate the effects of flooding on solar PV farm operation, focusing particularly on the effect on the insulation resistance of thin film PV modules after being of submerged in floodwaters.

The expected outputs of the study were: (i) an analysis of the effect on insulation resistance using both the dry and wet leakage current tests and the leakage voltage test, and (ii) to find if the leakage voltage test can be used as a faster and more convenient method for measuring insulation resistance on field.

The study also tried to find the threshold PV module conditions (after being subjected to flooded or wet conditions) that will still allow the operations of the solar PV farms within the required International Electrotechnical Commission (IEC) standards and avoid any loss opportunities and safety risks in the operation of the PVP system.

## Literature review

### Types of PV module failures

An overview on the types of PV module failures which occur after long-term outdoor exposure during their operation on the field have been explained by Rajput et al. [10]. The relationship between the type of materials of the module components and climate was studied by Omazic et al. [9]. The results of these two studies, which were discussed previously in the sub-section on “Effects of humid conditions on PV modules”, are summarized, and presented on Table 1. The information on Table 1 clearly shows that most of PV module failures or degradation are the results of the rapid increase in ambient humidity. These results support the reasons for conducting this study as discussed above.

**Table 1:** Summarize of PV module failure types [9, 10].

Module failure types	Stimulation/Climate conditions	External/Internal stresses	Effect on module
**Effect of EVA discoloration**	Ultraviolet radiation with high temperature (85°C—90°C or above).	External stress from UV and humidity. Internal stress from encapsulating material.	1–10%/year performance loss quantitative and qualitative reported.
**Effect of series & shunt resistance**	Radiation level: shunt resistance increases with decreasing the radiation level.	External stress from radiation level.	Efficiency decreases from influence of series and shunt resistance in PV module. Series resistance strongly affects PV performance compared to shunt resistance.
**Effect of corrosion**	High radiation, high ambient temperature, and humidity.	External stress from high radiation, high ambient temperature, and humidity. Internal stress from encapsulating material.	Conductivity of ethylene vinyl acetate (EVA) material decreases. Moisture ingress through EVA increasing and its effect on the metal part of module. Over than 90% of field failures caused from corrosion.
**Effect of hot spot**	High radiation.	External stress from high radiation. Internal stress from cell raw material and identify its reverse behaviour.	Performance degradation of copper indium gallium selenide (CIS) module over than 20% and amorphous silicon (a-Si) module 60%.
**Effect of de-lamination**	High ambient temperature and humidity.	External stress from high ambient temperature. Internal stress from material of the different additive layers such EVA and back-sheet.	More light reflection and the humid ingress through the front surface or either back surface of the module.
**Effect of bubble**	High ambient temperature and humidity.	Same as de-lamination.	Affected area smaller than de-lamination.
**Effect of crack in solar cell**	Vibration, applied loads and thermal effects (indirect stimulation by high radiation and ambient temperature.	Internal stress from module fabrication quality.	6% of solar cells cracks in fabrication process of a PV module. Potential to performance loss quantitative and qualitative.
**Effect of the bypass diode**	High ambient temperature and humidity.	Internal stress from module fabrication quality.	Potential to performance loss quantitative and qualitative.
**Effect of potential induced degradation (PID)**	High ambient temperature and humidity.	Internal stress from encapsulating properties.	Potential to performance loss quantitative and qualitative.

### Types of testing for PV module failures

Because of the constraints of on-site conditions only selected testing such as visual inspection and wet leakage current test can be done for the PV modules using the “IEC 61646 Standard” [11]. However, a high number of samplings are needed to have confidence on the results of the study.

The first test is the visual inspection test (IEC 61646 Standard), which is to detect any defects of the module. The usual major findings include broken, cracked, or split modules; bent parts of the modules; visible corrosion of any of the thin film layer of the modules (extending over more than 10% of any cell); bubble or delamination formation; and loss of mechanical integrity.

The second is the wet leakage current test, the purpose of which is to determine the insulation resistance of the module under wet operating conditions and verify that moisture from rain or any other humidity sources did not enter the active parts of the modules, where it might cause of corrosion, ground fault or safety risks. As a standard for areas bigger that 0.1 m^2^, the product of the measured insulation resistance and the module area should be greater than 40 MΩ.m^2^.

### Studies on leakage currents of PV modules

Leakage currents of PV modules lead to losses in the energy production of a PVP system. There are also added problems created by leakage currents such as safety risks, which are harmful to the PVP plant operators, to the PVP plant itself, and the distribution network. Most of the reports reviewed for this study showed how atmospheric climate conditions, particularly humidity, affect leakage currents from modules. High humidity and presence of other moisture sources increase the level of leakage currents of PV modules.

The humidity can have a significant impact on leakage current. Some orders of magnitude were shown in a test in the climate chamber laboratory, which accelerated the stress found in the PV field in a study done by Stephan H. and Michael K. [12]. They studied the temperature and humidity effects on the “potential induced degradation” (PID), which is the physical conditions of the degraded PV modules. Several levels of analysis were done. They first investigated the influence of temperature and humidity on the potential-induced leakage current. Then, they did a study consisting of an accelerated test method in a climatic chamber laboratory. Finally, they did a study involving field exposure under high voltage stress for two types of climates. The study showed that the PID was more strongly accelerated by stable load tests when regeneration in night-time was not considered.

RH can be a major cause in leakage current for both types of PV modules. Del Cueto J.A. and McMahon T.J. [13] has analysed the leakage current from crystalline silicon (c-Si) and amorphous silicon (a-Si) PV modules under high-voltage bias in field conditions. In high RH and wet conditions, the degree of leakage current was consistent with the conduction through the glass modules, which had notable leakages. It was reasonable for this to occur as conduction was through the wet top of the glass surface cover of the module that was bordered by the frame. On the other hand, at low RH, the degree of the activation energy and leakage current were more consistent with the conduction along the ethylene vinyl acetate (EVA)/glass interface where conductance is notable. In conclusion, surface and bulk glass conductivities were notable under wet conditions while under lower RH conditions, the combination of surface and bulk glass conductivity included EVA/glass surface conductivity control.

Surface soiling can result to higher leakage currents on soiled modules even at lower surface humidity. Michael K. and Stephan H. [14] studied the impacts of rain and soiling on the PID of the PV modules. Rainfall and soiling presented major hazards for PID, and studies on the factors for inducing high degree of leakage currents were conducted at two outdoor locations: at Freiburg (in Germany) for rainy conditions, and at Canary Island (in Spain) for conditions of heavy soiling by salty aerosols. Surface soiling can form within a few weeks or a few months, depending on the conditions of the surroundings. The outdoor leakage current increased with temperature, as shown by the Arrhenius law. The dependence on temperature was the same as the results of the laboratory tests that had the same activation energy. The relation of the grounding and leakage current on the surface conductivity given by moisture soiling and rain was clear. Interrupting the bias voltage for measuring the IV curve was a particularly useful technique for PID detection. Measurements at various times of day allowed the investigation of power losses at low light conditions, which was a more sensitive degradation indicator for early detection of PID.

There were no effects due to differences in the PV module mounting methods such as the clamp, backrail, and copper strap methods. The magnitude of leakage current still depended on the humidity. Voswinckel S. et al. [15] have studied the dependence of leakage current on the mounting techniques used for thin film a-Si modules. The goal of this study was to analyse leakage currents based on the difference of the mounting and grounding techniques of a-Si modules under outdoor operating conditions. Three distinctive conditions of humidity were recorded: high humidity and no dews on the modules, high humidity with dews, and humidity with rains. In this study, the module had only one strap of copper for simulating the module frame, hence the magnitude of the effect on a real module frame could have been bigger. With direct current (DC) flow, mounting with the backrail method was the most advantageous technique as, even if leakage current increased when water drops occurred, the increase was lesser than using clamps. With alternating current (AC) flow, clamp mounted modules showed the lowest leakage current, when the modules had no moisture. This was because the module clamp mounting area, which reduced the module ability, was much smaller than for the other mounting techniques.

As can be seen in these previous studies, humidity and other moisture sources increased the level of leakage currents of PV modules. The reports cited above show how atmospheric climate conditions, particularly humidity, affect leakage currents from modules.

Leakage currents of PV modules lead to losses in the energy production of a PV system. There are also added problems created by leakage currents that are harmful to the PV plant operators (such as safety risks), to the PV plants, and to related public assets. Hernández J.C. et al. [16] discussed the characteristic of insulation and leakage current in a PV system in relation to safety issues, and in-depth knowledge of active protective measures needed for the insulation electrical components of PV systems, especially under operating conditions with insulation faults. The equivalent circuit model that represented the insulation characteristics were obtained. This model can be applied for the evaluation of the insulation resistance of a PV system. The results can be used to analyse the potential risks to the public and used to design the best protection system. The paper discussed further the insulation behaviour of a PV system, and its reaction to meteorological parameters such as RH (relative humidity), module temperature, irradiance, wind speed, and atmospheric pressure, in both laboratory and field conditions. The study focused on those meteorological parameters that have greater influence on the PV system insulation, particularly the relationship between weather and insulation. It analysed the significant role that RH play in system insulation.

## Materials and methods

A set of submersible PV modules installed in a solar PV farm located in central region of Thailand (see in Fig 2A PV site study location), totalling of 15,792 thin film modules, was chosen for this evaluation. For orderly operational and management procedures, the PV farm was divided in two zones, however there were no technical difference between the two zones. The PV modules used had the following specifications (Table 2).

**Table 2:** PV modules specifications.

Parameters	Specifications
PV module type	Thin film
Peak power with tolerance	165 Wp of +5 W / 0 W
Maximum system voltage operating	1,000 V
Dimensions (L x W x H)	1,257 x 977 x 35 mm
Front cover of clear tempered glass	3.2 mm
Encapsulant	EVA and back sheet of weatherproof plastic film
Frame	Anodized aluminium alloy
Bypass diode	In protection junction box with ingress protection IP67 rating
Output cable	2.5 mm^2^ with 1,200 mm length, and multi-connector MC4 connectors

**Fig 3:**
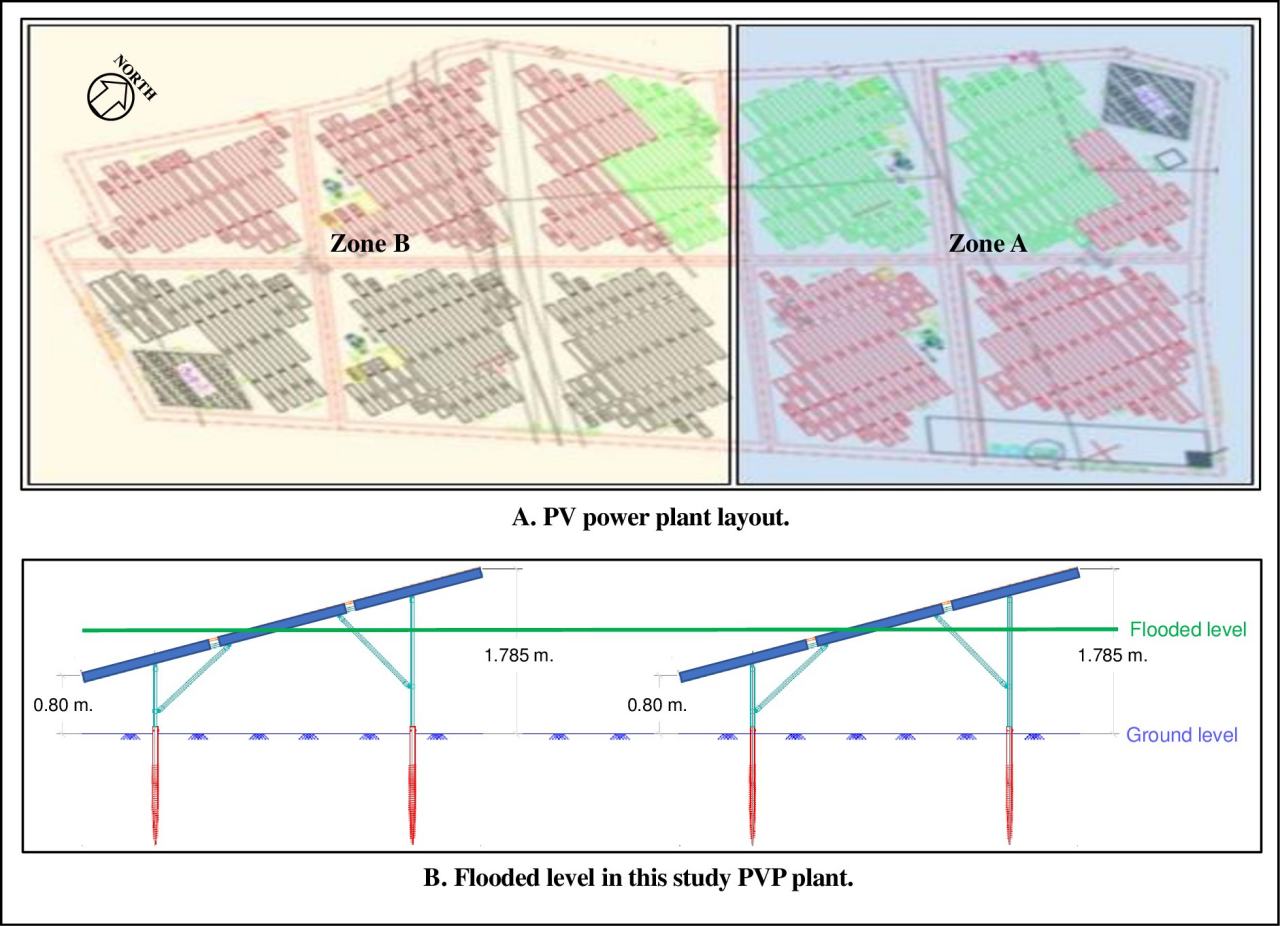
Layout of the PVP plant and the level of flooding. A. PV power plant layout. B. Flooded level in this study PVP plant.

Fig 3 shows the layout of the PVP plant studied and the level of flooding that occurred as recorded by the PVP plant operator. When the operator tried to do recovery operation of the plant after the flooding, an evaluation study showed abnormality; both Zones A and B had low degree of resistant insulation as detected by the inverter.

As soon as the floods subsided, the investigation and analysis of the performance of the PV modules were given high priority, so that the PV power system can fully recover and work to minimize energy production losses.

**Fig 4:**
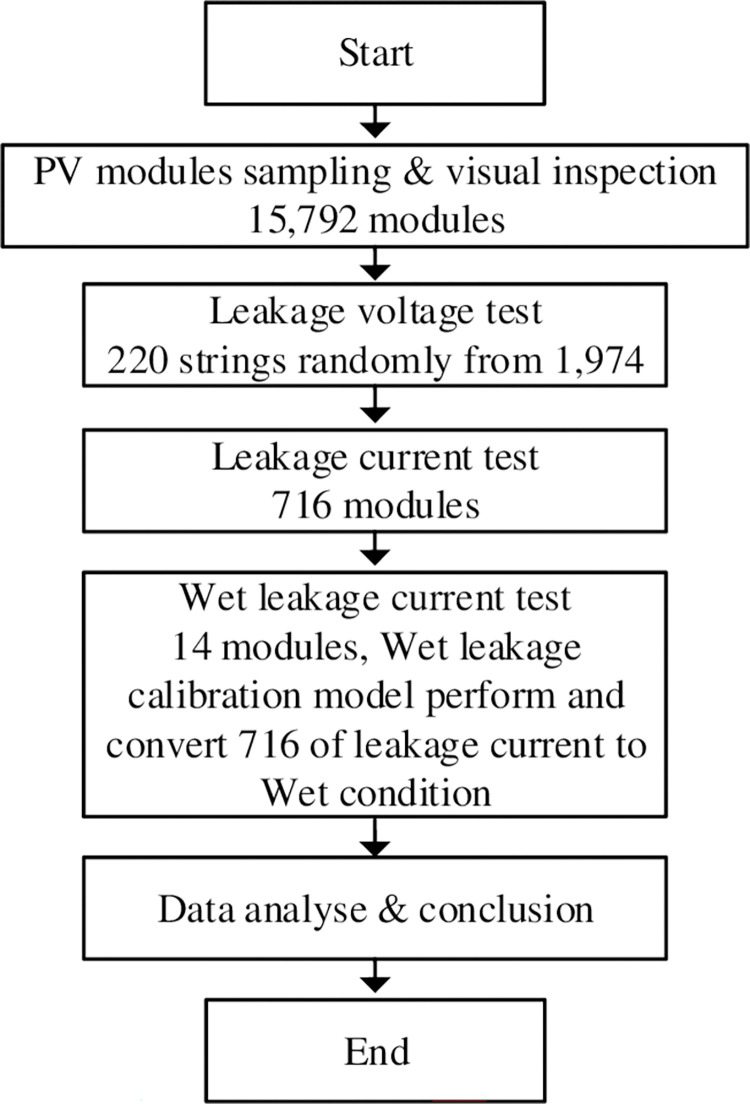
Testing procedure and number of samplings of each test.

To minimize opportunity losses, technical losses, safety risks and time delays, a PV module test method based on basic instruments and measurement techniques had to be developed. With enormous number of modules, long investigation period, and difficult field conditions, only three types of tests were conducted to evaluate the status of the modules. The tests included measurement of leakage voltage of the PV system, leakage current (in terms of insulation resistance) of the PV modules, and wet leakage current (in terms of insulation resistance) of the PV modules (see Fig 4).

The test was to estimate the number (in %) of modules with insulation resistance below the requirements of IEC 61646 standard [11] and further, to find any correlation between the measured leakage voltage and the insulation resistance of the PV modules. The possible increase in leakage current and leakage voltage study was also investigated. The details of the study are described in next sections.

### Sampling

All the modules from Zones A and B were examined for any visual defects or damage before sampling for testing was done. They were all found to be undamaged.

For the purpose of testing leakage voltage, 220 strings were chosen randomly from the test population. According to Krejcie & Morgan Table [17], 320 strings were needed but due to field constraints, only 220 strings were selected out of 1,974. As mentioned before, this study was conducted for a PV solar farm that was submerged by floodwaters and as such, many PV string locations were difficult to access and field measurements cannot be done under proper testing conditions. This included limitation in the operation time for measurements to minimize testing costs and plant income losses (due to operation interruptions). To make sure that the samples were representative, random sampling was conducted covering all areas of each zone.

For leakage current measurements, the whole module population of the selected group (i.e., 15,792 modules) was considered for sampling and the required number of samples [see again reference 17] came out to be 377. However, for a deeper investigation and higher confidence, 716 (nearly double) modules were tested.

For wet leakage current (wet insulation resistance) testing; to minimize and avoid damage on the sample modules during testing, 14 modules, covering all ranges of insulation resistance (dry) were chosen from the 716 modules already tested for dry insulation resistance. The results of these tests were used to calculate the wet insulation resistance of the 716 modules which were also individually tested for insulation resistance in dry conditions.

Samples tested was only about 4% of the total number of modules. The sample size was small because of constraints due to time and cost, as mentioned earlier.

### Leakage voltage measurement

**Fig 5:**
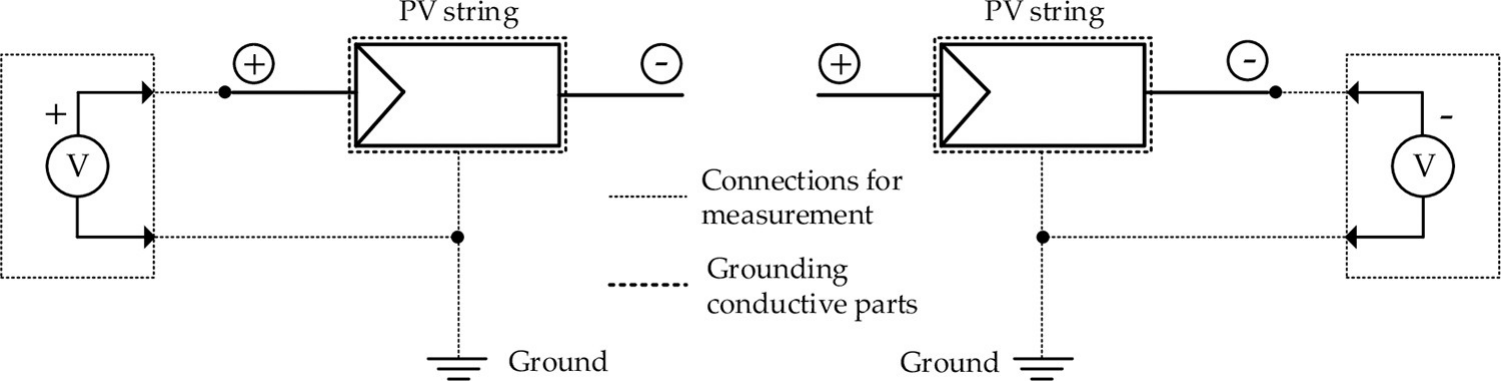
Measuring of leakage voltage of PV strings.

Voltage was measured at string level instead of single module level keeping in view the fact that the voltage at module level was extremely low as compared with that at string level. A duly calibrated Fluke 376 clamp meter with a measurement tolerance of ±1% was used to measure the leakage voltage. Circuits under test were disconnected from the rest of the system before the measurement. The DC voltage was measured between the positive and negative terminals of the module and the ground (or the module frame of the mounting structure). Measurements were done on clear days between 09:00AM and 04:00PM (see Fig 5) [11, 16, 18, 19].

### Dry leakage current measurement

**Fig 6:**
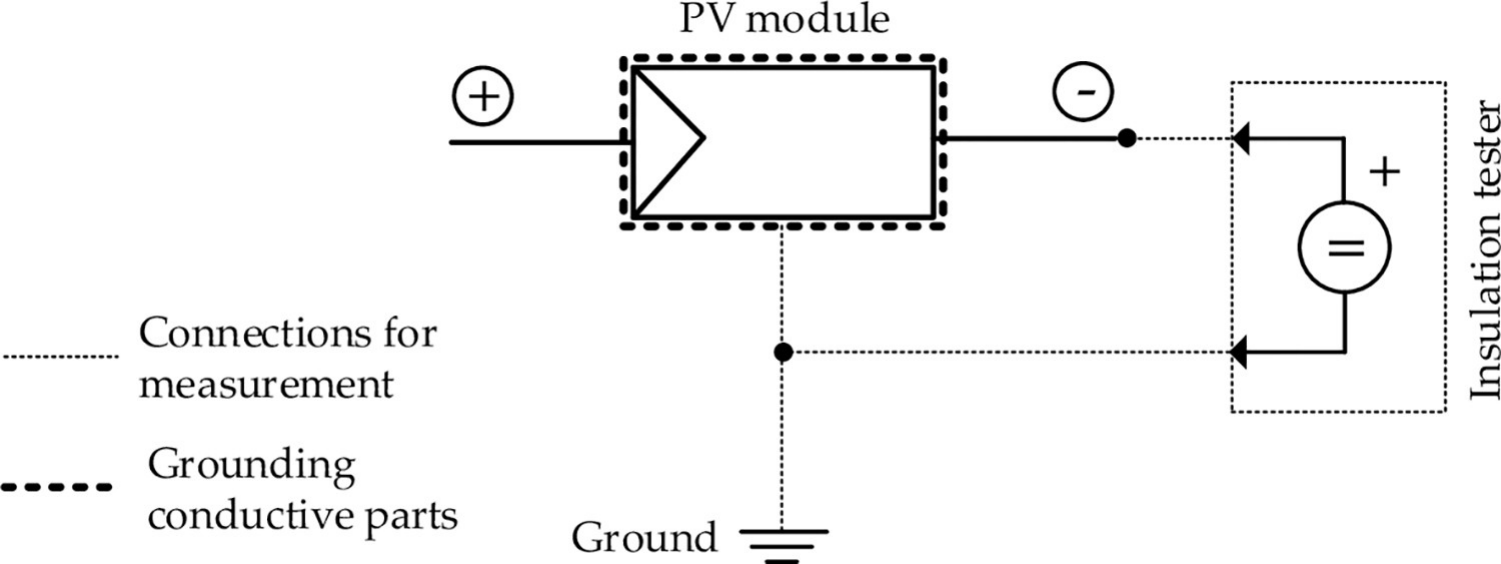
Measuring of leakage current of PV modules.

Insulation resistance was taken as a measure of the leakage current of the PV modules. Each sampled module was disconnected from the rest of the system and insulation resistance was measured generally in accordance with the IEC 61646 standard [11] but performed in dry condition, and with some minor variations as per field conditions. A HIOKI 3118 MΩ Hi tester with a measurement tolerance of ±5% was used to measure insulation resistance. The maximum system voltage of the modules under test was 1,000 VDC. Insulation was measure between the negative terminal of the modules and the ground frame (see in Fig 6).

### Wet leakage current measurement

Wet insulation resistance measurement was performed following the IEC 61646 standard [11]. Water was used as the wetting agent and modules were placed in a water filled container after removing them from the arrays. A 1,000 VDC was applied between the module terminals and the water using a HIOKI 3118 MΩ Hi tester. The maximum system voltage for single module as mentioned earlier was 1,000 VDC. The calibration model formula was developed through a sampling test of 10 category groups of dry leakage current per one wet leakage current group. Later, the measurement of wet leakage current of 716 modules was done using the developed formula.

## Results and discussions

Summaries of the results for all three types of tests conducted are given in the following sub sections (see also Table 3 below). It is important to mention again that wet insulation resistance was measured for a limited number of modules due to operational constraints. Based on these measured results, wet insulation resistance was calculated for 716 modules.

**Table 3:** Summary of test results.

S.#	Test Type	No. of Samples	Results
1	Leakage voltage test	220	Leakage voltage range 7 to 219 V Average leakage voltage 46 V
2	Leakage current test (Insulation resistance dry)	716	Insulation resistance range 15 to higher than 150 MΩ Average insulation resistance 75 MΩ
3	Wet leakage current test (Insulation resistance wet)	14	Insulation resistance range 2 to 60 MΩ Average insulation resistance 26 MΩ

### Leakage voltage test

**Fig 7:**
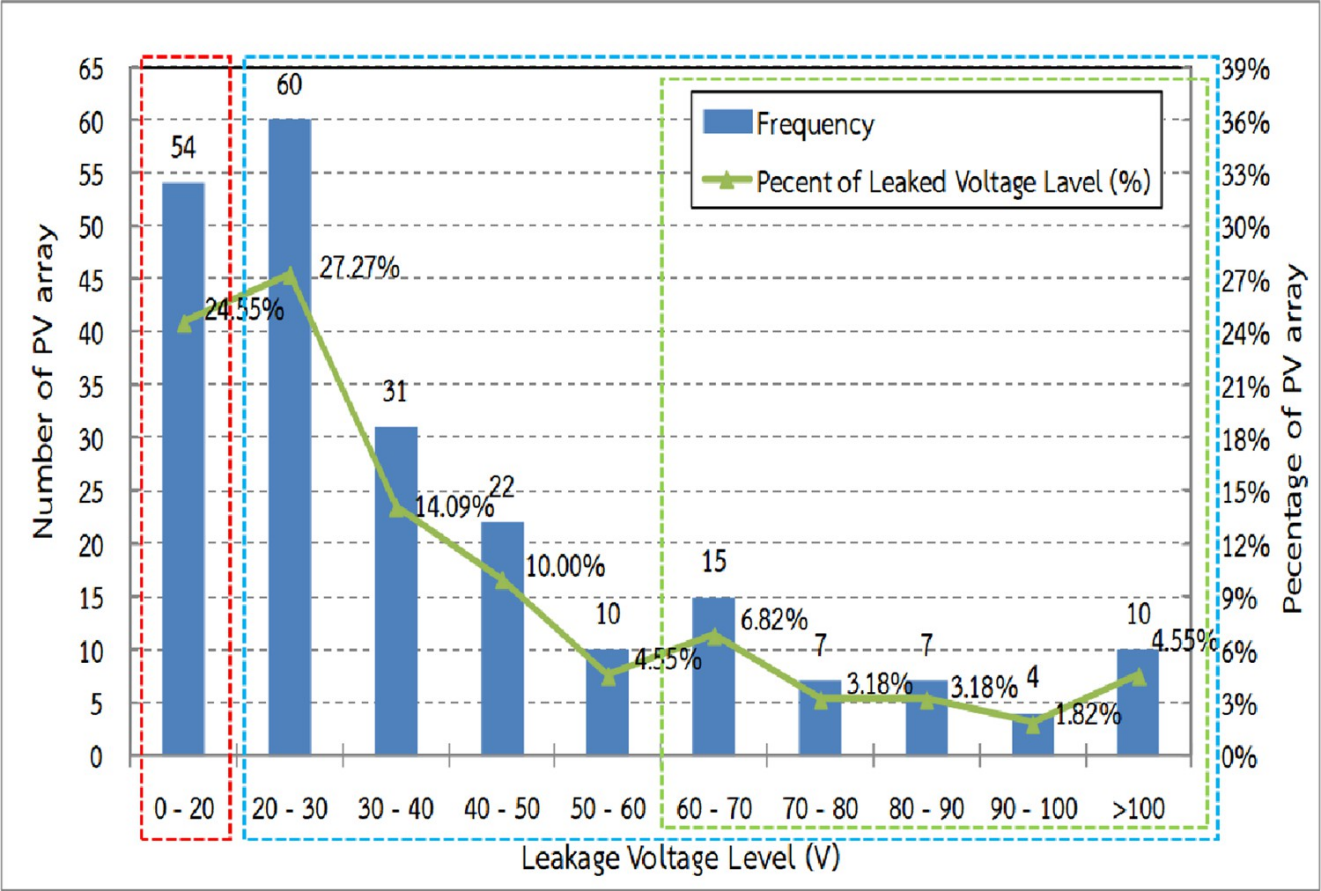
Leakage voltage distribution of sampled strings.

A total of 220 strings were tested. Each string was formed by connecting 8 modules in series. The results showed leakage voltage variation from 7 to 219V averaging to 46 V for both “positive pole to ground” and “negative pole to ground”. As shown in Fig 7, 24.55% (red dash) of sampled strings had a leakage voltage of below 20 V which was quite normal. However, 75.45% (indicated by the blue dash) of sampled strings showed higher values. This can affect the inverter operation as a leakage voltage higher than 20 V can cause an impedance lower than the requirements. Moreover, 25.90% (green dash) of strings showed leakage voltage of more than 60 V, which can result in halting of the operation of inverters. The main cause of leakage voltage was possibly from the reduction of insulation resistant of PV modules due to high humidity. This probably diffused into the modules during its submersion in the water. This was consistent with the findings of Stephan H., Del Cueto J.A. and Michael K. [12–14].

In the case of leakage voltage over than 100 V, it was possible that the reduction of insulation resistance of the modules and string cable were also included because the flood waters covered all parts of the PV string.

### Dry leakage current test

**Fig 8:**
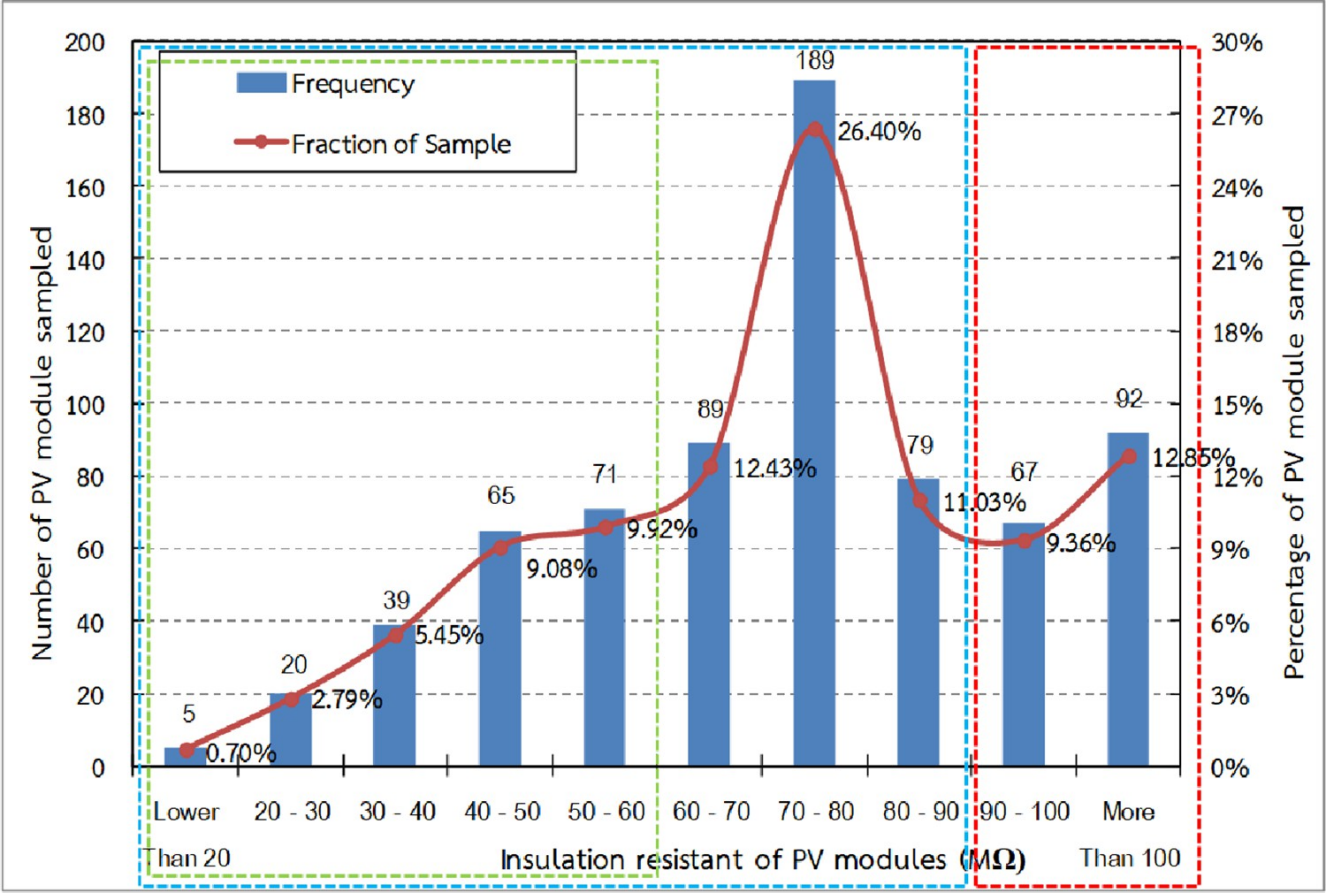
Insulation resistance distribution of the sampled PV modules.

The constraints in using the IEC 61646 leakage current test, performed under wet test condition, were explained previously. This study modified the test method by measuring the leakage current in dry condition to determine the trend in the reduction of the insulation resistance of the PV modules, and the effect of this reductio on the PVP system operation. The insulation resistance of the sampled PV modules was found to vary from 15 to 231 MΩ with an average insulation resistance value of about 75 MΩ. The insulation resistance distribution of the sampled PV modules is presented in Fig 8. 159 sampled PV modules, or 22.21% (red dash) of the total PV modules sampled, had insulation resistance higher than 90 MΩ that did not affect the inverter operation. 557 sampled PV modules, or 77.79% (blue dash) of the total sampled PV modules, had insulation resistance lower than 90 MΩ, which could have interrupted inverter operation. 200 modules, or 27.93% (green dash) of the sampled PV modules, had insulation resistance lower than 60 MΩ that could have dropped to below 40 MΩ (the threshold for this specific size of module) in wet conditions, resulting in halting of inverter operation.

The results showed the same trend as for leakage voltage, confirming an inverse proportional relationship between the voltage and insulation resistance. It is possible to interfere in the inverter operation to find and confirm the module leakage current test results. The insulation equivalent circuit for the PV module proposed by Hernández J.C. et al. [16] was used to analyze the insulation resistance of the PV array that was connected to the inverter. Sitthiphol N. et al. [20] also applied this proposed equation for finding the PV modules and strings insulation resistance in their study. When the PV modules were connected in series and parallel to be the PV array, the equivalent circuit was also connected in the same pattern.

The important parameter of the insulation equivalent circuit R_s_ is the series insulation resistance of the PV module (Ω), wherein R_p_ is parallel insulation resistance of the PV module (Ω), R_iso_ is PV array insulation resistance (Ω), m is the number of strings in the array, and n is the number of PV module that are series connected in the string. Hence, the PV array insulation resistance can be calculated from the following equations.


$$R_{iso}=(R_{s}+R_{p})/(m\;x\;n)$$ (1)

In the case that (R_s_ + R_p_) is constant in each module.


$$1/R_{iso}=1/(R_{s1}+R_{p1})+1/(R_{s2}+R_{p2})+\ldots 1/(R_{s(m\;x\;n)}+R_{p(m\;x\;n)})$$ (2)

In the case that (R_s_ + R_p_) is different in each module.

From the insulation resistance data of the sampled PV module, the measured data is the value of (R_s_ + R_p_) and the insulation resistance of PV array that are connected to the inverter can be estimated by using this formula.

The PV array consisting of 3,700 PV modules had an estimated insulation resistance of about 10.67 kΩ. This was a little higher than the minimum input impedance between the inverter input and the ground at 10 kΩ, as recommended in the inverter installation manual. However, this estimation did not include other effects such as cable insulation resistance. The PV array insulation resistance was lower than 10 kΩ when every effect was included.

New PV modules usually have insulation resistant about 150 MΩ or higher. The insulation resistance of the PV array, which consisted of 3,700 modules, was 40.54 kΩ, which was 4 times higher than the minimum input impedance between inverter input and ground. This showed that PV arrays consisting of the new PV modules can work normally with the inverter. Thus, the PV modules in both the A and B zones have lower insulation resistance than the normal PV modules. A PV array insulation resistance lower than 10 kΩ can potentially affect the operation of the PV inverter.

### Wet leakage current test

According to IEC 61646 Standard, a minimum of 40 MΩ.m^2^ insulation resistance is required for a PV module with an area of greater than 0.1 m^2^. Thus, in this specific case, a minimum of 40 MΩ insulation resistance is needed to meet the criterion of the standard.

**Fig 9:**
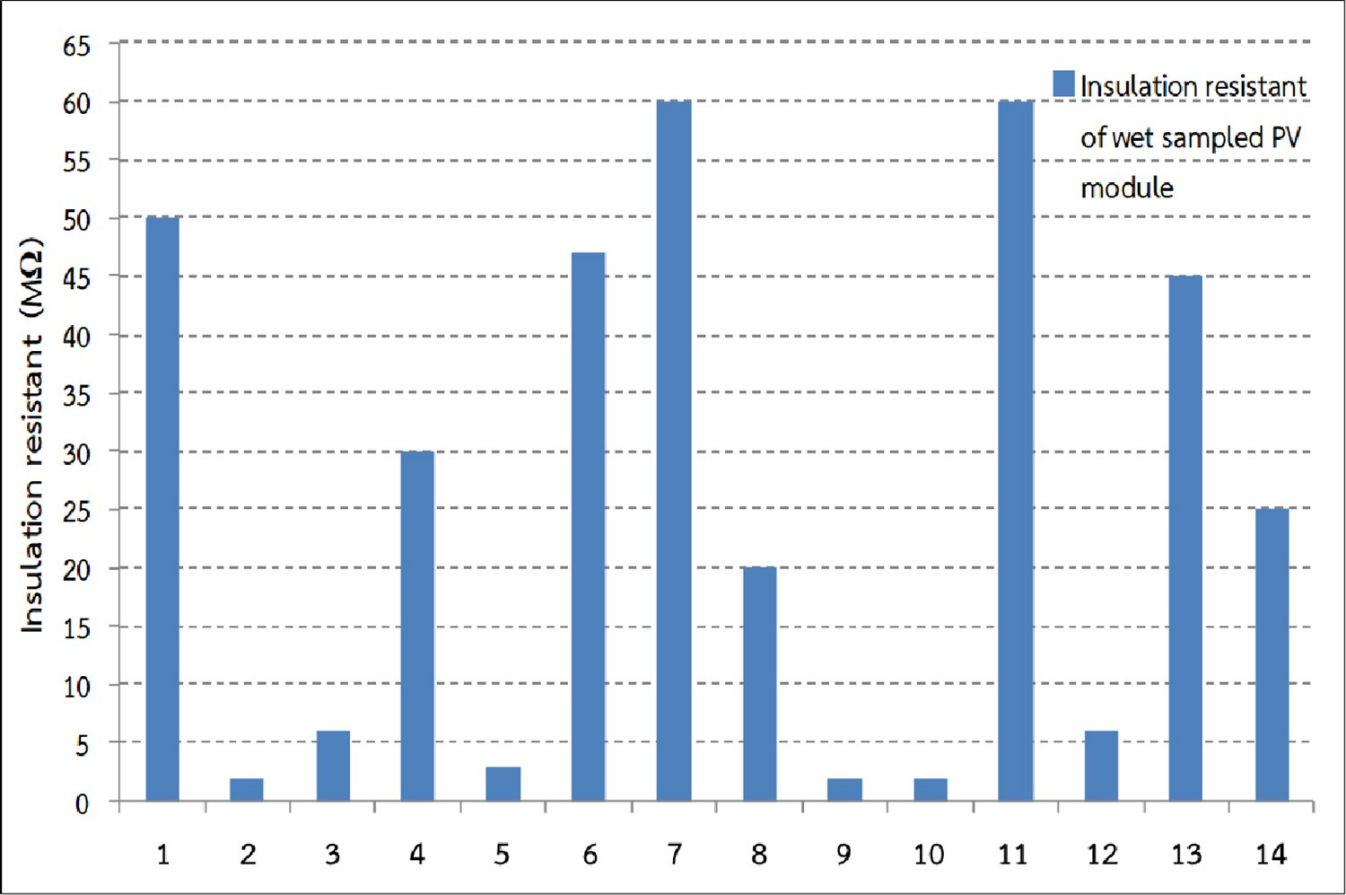
Insulation resistance of wet sampled PV module.

The insulation resistance of each sampled wet PV module (representing all measured ranges of dry insulation resistance) is displayed in Fig 9 (S2 Data). The measured insulation resistance of the sampled wet PV modules varies from 2 to 60 MΩ with an average insulation resistance of about 26 MΩ. Although, the sample size of this test is quite small, the various technical and cost constraints have been investigated and well covered. About 64% of the samples, represented the lower level of insulation resistance compared to the standard. This figure should be enough to justify the trend of modules insulation resistance for this PVP plant resulting from this study.

**Fig 10:**
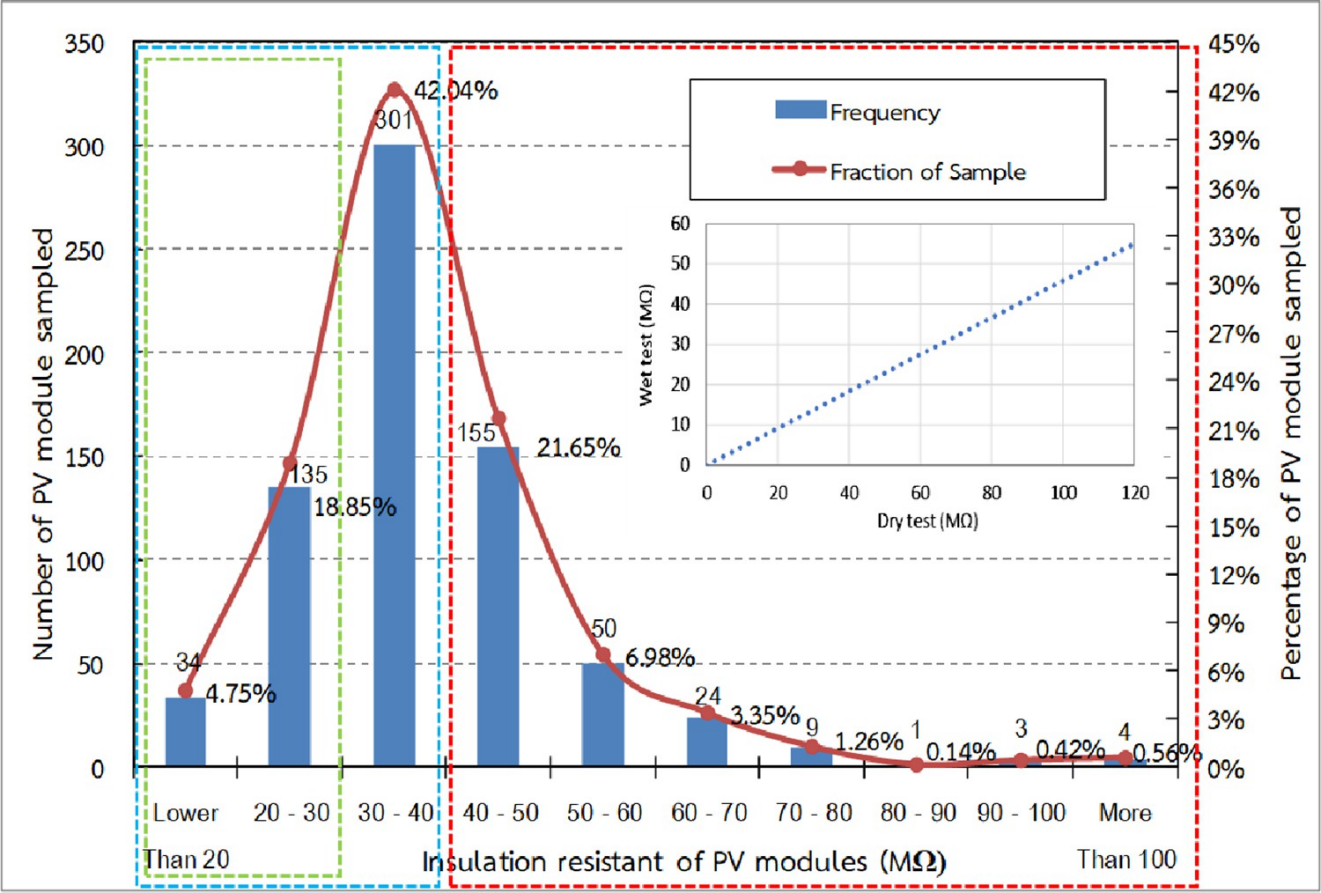
Insulation resistance of wet sampled PV module.

The calculated resistance of the wet insulation for 716 samples using the results of representative modules shows that insulation resistance varied from 11 to 110 MΩ with an average of 39 MΩ. The distribution of the insulation resistance of the sampled wet PV modules is shown in Fig 10.

246 wet PV module samples or 34.36% (red dash) of the total wet PV module samples had insulation resistance higher than 40 MΩ, which followed the requirement of IEC 61646 standard. However, 470 wet PV module samples or 65.64% (blue dash) of the total samples had insulation resistance lower than 40 MΩ, which did not follow the requirement of IEC 61646 standard, and this could possibly interrupt the inverter operation. Moreover, 169 of wet PV module samples or 35.96% (green dash) had insulation resistance lower than 30 MΩ, which could have stopped the inverter operation as per set point in this system.

**Fig 11:**
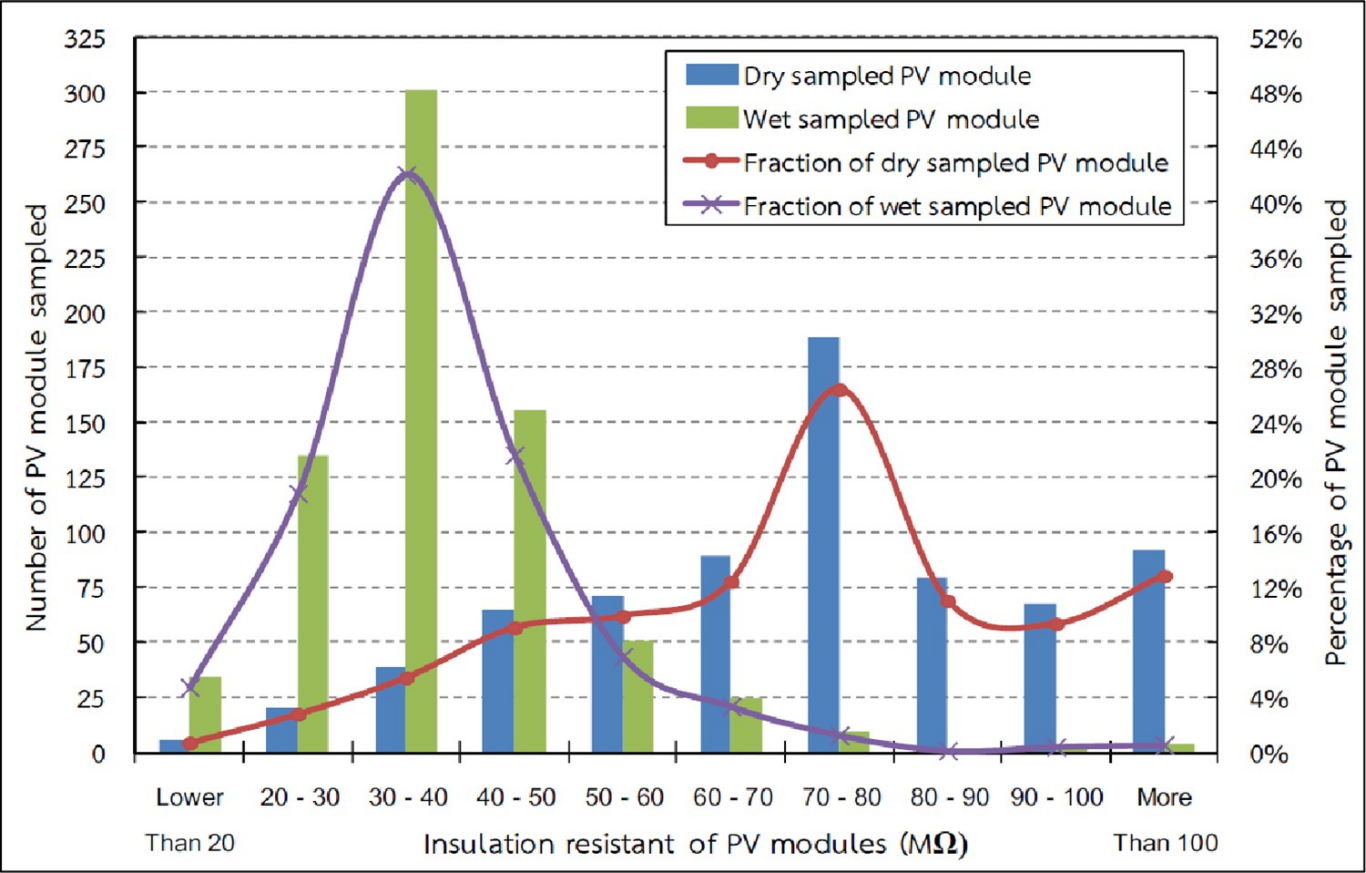
Relationship between the wet leakage current test and dry leakage current test.

These test results showed the same trend as the results of the dry leakage current test, which proved that there is a direct proportional relationship between the results of the wet leakage current test and dry leakage current test of the PV modules (Fig 11).

## Conclusions

Measurement of dry leakage current and wet leakage current of the PV module insulation resistance require special instruments and complicated procedures in field conditions. These result to longer time and higher cost of testing. An alternative test that allows simpler instruments and procedure to give an indication of the PV module insulation resistance is the leakage voltage test.

Nearly the same trend was recorded for the test results for both leakage voltage and leakage current. Leakage voltage tests can provide an indication of the level of PV module insulation quality with acceptable accuracy. The leakage voltage test results have the same trend as both the leakage current and wet leakage current tests. In addition, the testing time and cost needed in the leakage voltage test are lower than leakage current and wet leakage current tests. Thus, the leakage voltage test is suitable for testing the insulation quality of exceedingly high number of PV modules. However, for highly accurate and precise results, wet leakage current test is still recommended.

Finally, in finding the threshold PV module conditions (after being subjected to flooded or wet conditions) that can still allow their operations within the required IEC standards, the following were the results of the three methods of testing that were conducted. Insulation resistance of the PV modules dropped significantly if compared with new unused modules (on average 50%). The reduction of the insulation resistance of PV modules resulted in leakage current and cause disruptions and losses. A significant part of sampled modules (66%) did not pass the minimum criterion of IEC 61646 standard for wet insulation resistance testing, wherein there were deep moisture ingress in the modules.

A reasonably high number of modules were found with low insulation resistance and high leakage voltage values. High leakage voltage creates safety hazards [16]. This can interrupt the inverter operation even with a slight variation in climatic conditions thus compromising the reliability of the electricity supply.

## Supporting information

S1 Data (XLSX)

S2 Data (XLSX)
